# Supplementary material for: Brain network differences in second language learning depend on individual competencies
Source: Imaging Neurosci (Camb). 2026 Sep 30;4:IMAG.a.1379. doi: 10.1162/IMAG.a.1379 (PMC13628499; doi:10.1162/IMAG.a.1379)
Supplement: Supplementary file 1 [file IMAG.a.1379_supp.pdf]

Supplementary Material

Title of Manuscript: Brain Network Differences in Second Language Learning Depend on Individual Competencies

Supplementary Methods

Stimulus Validation and Exclusions

Four items contained typographical errors. Three of these affected all French-speaking participants and were excluded from all analyses. In a fourth case, the Finnish word *huili* (instead of *huilu*, “flute”) was presented during the intervention phase for four German-speaking participants, while *huilu* was used in the test phases. Due to this inconsistency, data for this specific word were excluded for these participants. Items with typographical errors are marked with an asterisk (*) in *Supplementary Table S1*.

Additionally, nine Finnish words used in the experiment did not correspond exactly to the intended German source words due to translation errors during stimulus preparation (marked with a dagger [†] in the Supplement). These translation discrepancies are detailed in the Supplementary Table and Table Note. All translation errors were present in the stimuli as presented to participants. Since none of the participants had prior knowledge of Finnish, these errors are not expected to have affected task performance or interpretation of results.

Table S1

Full Stimulus Characteristics for All Items

**German (syll./len./Zipf)**	**French (syll./len./Zipf)**	**Finnish (syll./len./Zipf)**	**English (translation)**
Vogel (2/5/4.45)	oiseau (2/6/4.31)	lintu (2/5/4.31)	bird
Hund (1/4/4.86)	chien (2/5/4.86)	koira (2/5/5.04)	dog
Katze (2/5/4.49)	chat (1/4/4.76)	kissa (2/5/4.6)	cat
Wolf (1/4/4.57)	loup (1/4/4.36)	susi (2/4/4.23)	wolf
Esel (2/4/3.91)	âne (2/3/3.8)	aasi (2/4/3.67)	donkey
Kuh (1/3/4.04)	vache (2/5/4.14)	lehmä (2/5/4.04)	cow
Schaf (1/5/3.73)	mouton (2/6/3.92)	lammas (2/6/3.85)	sheep
Hirsch (1/6/3.85)	cerf (1/4/3.78)	peura (2/5/3.6)	deer
Hahn (1/4/4.29)	coq (1/3/3.83)	kukko (2/5/3.78)	rooster
Wal (1/3/3.46)	baleine (3/7/3.73)	valas (2/5/3.26)	whale
Affe (2/4/3.72)	singe (2/5/3.93)	apina (3/5/3.83)	monkey
Rabe (2/4/3.42)	corbeau (2/7/3.49)	korppi (2/6/3.45)	raven
Schlange (2/8/4.11)	serpent (2/7/4.02)	käärme (3/6/3.71)	snake
Fliege (2/6/3.81)	mouche (2/6/3.9)	lentää (2/6/4.62) †	fly
Ente (2/4/3.78)	canard (2/6/4.17)	ankka (2/5/4.07)	duck
Donner (2/6/3.57)	tonnerre* (3/6/3.81)	ukkonen (3/7/3.57)	thunder
Regen (2/5/4.63)	pluie (2/5/4.56)	sade (2/4/4.16)	rain
Wind (1/4/4.69)	vent (1/4/4.8)	tuuli (3/5/4.57)	wind
Fluss (1/5/4.44)	fleuve (2/6/4.32)	joki (2/4/4.01)	river
Schnee (1/6/4.45)	neige (2/5/4.56)	lumi (2/4/4.17)	snow
Tropfen (2/7/4.15)	goutte (2/6/4.03)	kihti (2/5/2.7) †	drop
Adler (2/5/4.19)	aigle (2/5/3.82)	kotka (2/5/4.28)	eagle
Eule (2/4/3.5)	hibou (2/5/3.23)	pöllö (2/5/3.36)	owl
Henne (2/5/3.38)	poule (2/5/4.21)	kana (2/4/4.09)	hen
Boot (1/4/4.52)	bateau (2/6/4.61)	vene (2/4/4.23)	boat
Füller (2/6/3.09)	stylo (2/5/3.85)	kynä (2/4/3.89)	pen
Ball (1/4/4.64)	balle (2/5/4.6)	pallo (2/5/4.63)	ball
Dose (2/4/3.91)	canette* (2/7/3.21)	tölkit (2/6/2.8) †	can
Hammer (2/6/4.4)	marteau (2/7/3.87)	vasara (3/6/3.75)	hammer
Schere (2/6/3.78)	ciseaux (2/7/3.54)	sakset (3/6/3.57)	scissors
Tafel (2/5/4.17)	tableau (2/7/4.82)	lauta (3/5/3.51) †	board
Tür (1/3/4.9)	porte (2/5/5.47)	ovi (2/3/4.48)	door
Flugzeug (2/8/4.46)	avion (2/5/4.86)	kone (2/4/4.84) †	airplane
Foto (2/4/5.02)	photo (2/5/5.14)	kuva (2/4/5.43)	photo
Korken (2/6/3.01)	bouchon (2/7/3.79)	pistoke (3/7/2.51) †	cork
Rakete (3/6/3.89)	missile (3/7/3.84)	ohjus (3/5/3.79)	rocket
Feder (2/5/3.91)	ressort (3/7/4.25)	kevät (2/5/4.43) †	spring
Säge (2/4/3.37)	scie (1/4/3.62)	näin (2/4/6.04) †	saw
Pfeife (2/6/3.6)	sifflet (2/7/3.39)	pilli (2/5/3.38)	whistle
Trommel (2/7/3.55)	tambour (3/7/3.65)	rumpu (2/5/3.23)	drum
Hupe (2/4/3.09)	klaxon (2/6/3.01)	tuutata (3/7/1.53) †	horn
Teller (2/6/4.09)	assiette (3/8/4.07)	lautanen (3/8/3.44)	plate
Schwert (1/7/4.1)	épée (2/4/4.2)	miekka (3/6/3.84)	sword

**German (syll./len./Zipf)**	**French (syll./len./Zipf)**	**Finnish (syll./len./Zipf)**	**English (translation)**
Kanone (3/6/3.44)	canon (2/5/4.36)	tykki (2/5/3.7)	cannon
Besen (2/5/3.49)	balai* (2/6/3.36)	luuta (2/5/3.48)	broom
Geld (1/4/5.59)	argent (2/6/5.37)	raha (2/4/4.86)	money
Flöte (2/5/3.54)	flute (2/5/3.65)	huilu* (2/5/3.15)	flute

Note. Syll. = number of syllables; len. = length in letters; Zipf = Zipf frequency. English translations are provided for illustration only; only the original items were used as experimental stimuli. Words marked with an asterisk (*) or a dagger (†) are described in detail in the Supplementary Methods.

Table S2

Vocabulary Test: Effects of Learning Strategies on Post-Learning Scores

**Predictor**	***F(df1, df2)***	***p***	η^2_g
Performance Group	> 86.80 (1, 79–81)	< .001	0.45–0.56
Retrieval Practice	0.06 (1, 81)	.807	0.00
Corrective Feedback	10.95 (1, 80)	.001	0.03
Multisensory Learning (Encoding)	0.81 (1, 81)	.372	0.00
Multisensory Learning (Retrieval)	0.01 (1, 81)	.905	0.00
Distributed Learning	2.53 (1, 79)	.116	0.03

Note. $F(1, 81)$ refers to the F-statistic with degrees of freedom (*df1, df2*). *df1* indicates degrees of freedom numerator. *df2* indicates degrees of freedom denominator. p = significance level. η^2_g = generalized eta squared, indicating effect size.

Table S3

Effects of Performance Group and Session on d' in the Translation Recognition Task

**Predictor**	***F(df1, df2)***	***p***	η^2_g
Session	1603.27 (1, 75)	< .001	.89
Performance Group	30.16 (1, 75)	< .001	.20
Session × Performance Group	37.51 (1, 75)	< .001	.16
Retrieval Practice	0.00 (1, 75)	.988	.00
Corrective Feedback	3.16 (1, 74)	.080	.01
Multisensory Learning (Encoding)	0.03 (1, 75)	.874	.00
Multisensory Learning (Retrieval)	0.36 (1, 75)	.551	.00
Distributed Learning	0.12 (1, 73)	.727	.00

Note. $F(1, 75)$ refers to the F-statistic with degrees of freedom *(df1, df2)*. *df1* indicates degrees of freedom numerator. *df2* indicates degrees of freedom denominator. p values are two-tailed. η^2_g = generalized eta squared effect size measure.

Table S4

Effects of Performance Group and Session on Criterion in the Translation Recognition Task

**Predictor**	***F(df1, df2)***	***p***	η^2_g
**Performance Group**	5.92 (1, 75)	.017	.04
**Session**	9.49 (1, 75)	.003	.05
Performance Group × Session	0.75 (1, 75)	.390	.00
Retrieval Practice (RP)	0.07 (1, 75)	.788	.00
Performance Group × RP	0.07 (1, 75)	.794	.00
Corrective Feedback	1.26 (1, 74)	.265	.00
Performance Group × CF	0.29 (1, 74)	.590	.00
Multisensory Learning at Encoding (EM)	0.59 (1, 75)	.443	.00
Performance Group × EM	0.25 (1, 75)	.620	.00
Multisensory Learning at Retrieval (RM)	1.76 (1, 75)	.189	.00
**Performance Group × RM**	4.67 (1, 75)	.034	.01
Distributed Learning (DL)	0.40 (1, 73)	.528	.01
**Performance Group × DL**	5.40 (1, 73)	.023	.07

*Note***.** *F*(1, 75) refers to the F-statistic with degrees of freedom *(df1, df2)*. *df1* indicates degrees of freedom numerator. *df2* indicates degrees of freedom denominator. *p* values are two-tailed. η^2_g = generalized eta squared effect size measure. Bold highlighted predictors represent significant effects.

Table S5

N400 Amplitude Difference (Incongruent Minus Congruent) by Performance Group, Session, and Learning Strategy

Model	Predictor	*F(df1, df2)*	*p*	η^2_g
**Main Analysis**	Performance Group	14.44 (1, 75)	.000	11
	Session	99.52 (1, 75)	.000	.32
	Performance Group × Session	6.79 (1, 75)	.011	.03
**Retrieval Practice**	Performance Group	19.73 (1, 75)	.000	.13
	Retrieval Practice (RP)	0.00 (1, 75)	.978	.00
	Performance Group × RP	0.74 (1, 75)	.394	.00
**Corrective Feedback**	Performance Group	12.40 (1, 74)	.001	.08
	Corrective Feedback (CF)	0.01 (1, 74)	.927	.00
	Performance Group × CF	0.15 (1, 74)	.703	.00
**Multisensory Learning (Encoding)**	Performance Group	18.86 (1, 75)	.000	.14
	Encoding (EM)	0.03 (1, 75)	.861	.00
	Performance Group × EM	3.65 (1, 75)	.060	.02
**Multisensory Learning (Retrieval)**	Performance Group	17.12 (1, 75)	.000	.13
	Retrieval (RM)	0.00 (1, 75)	.961	.00
	Performance Group × RM	0.00 (1, 75)	.967	.00
**Distributed Learning**	Performance Group	15.81 (1, 73)	.000	.18
	Distributed Learning (DL)	0.30 (1, 73)	.588	.00
	Performance Group × DL	0.11 (1, 73)	.745	.00

Note. All models by learning strategies tested post-learning N400 differences (incongruent – congruent). $F(1, 75)$ refers to the F-statistic with degrees of freedom *(df1, df2)*. *df1* indicates degrees of freedom numerator. *df2* indicates degrees of freedom denominator. *p* values are two-tailed. η^2_g = generalized eta squared effect size measure.

Table S6

Topographic Map Preponderance by Map, Session, Congruency, and Performance Group

Model	Predictor	*F(df1, df2)*	*p*	η^2_g	*Epsilon*
**Epoch 1 (TF 0–73)**	Map	8.35 (1, 75)	.005	.08	
**Epoch 2 (TF 73–160)**	Performance Group × Map	3.40 (1.74, 130.52)	.043	.03	0.87
	Session × Map	17.67 (1.90, 142.69)	< .001	.05	0.95
	Congruency × Map	17.67 (1.78, 133.20)	< .001	.02	0.89
	Session × Congruency × Map	16.48 (1.97, 147.59)	< .001	.01	0.98
**Epoch 3 (TF 160–199)**	Map	21.93 (1, 75)	< .001	.12	
	Performance Group × Map	5.18 (1, 75)	.026	.03	
	Session × Map	6.32 (1, 75)	.014	.02	
	Congruency × Map	8.13 (1, 75)	.006	.02	
	Session × Congruency × Map	15.63 (1, 75)	< .001	.02	

Note. $F(1, 75)$ refers to the F-statistic with degrees of freedom *(df1, df2)*. *df1* indicates degrees of freedom numerator. *df2* indicates degrees of freedom denominator. Epsilon indicates Greenhouse-Geisser multiplier for degrees of freedom, *p*-values and degrees of freedom in the table incorporate this correction. *p* values are two-tailed. η^2_g = generalized eta squared effect size measure.

Figure S1

Permutation p value Trajectories Over Time for Significant Global pAUC Tests

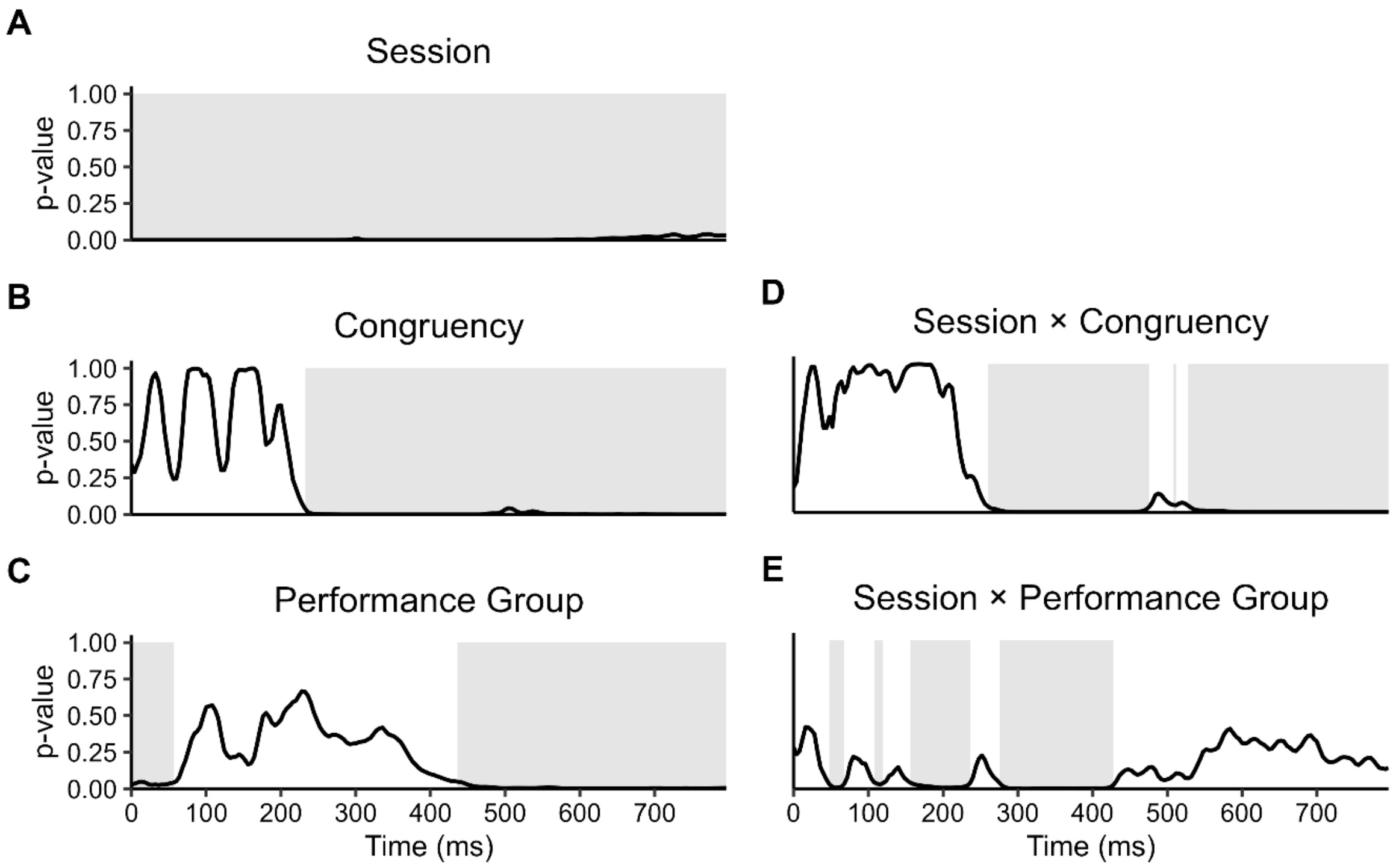

Note. Each panel (A–E) shows the permutation *p* value over time for the corresponding global pAUC test across the 0–796 ms epoch. Panels A–C (left column) display main effects of Session, Congruency, and Performance Group; panels D–E (right column) display the Session × Congruency and Session × Performance Group interactions. Gray shading indicates time windows with $p < .05$.
